# Web-based, rapid and contactless management of ambulatory patients for SARS-CoV-2-testing

**DOI:** 10.1186/s12879-021-06249-7

**Published:** 2021-06-07

**Authors:** Jannik Stemler, Oliver A. Cornely, Torsten Noack-Schönborn, Corinna Fohrholz, Sofie Schumacher, Leonard Poluschkin, Bernd Binder, Clara Lehmann, Georg Langebartels

**Affiliations:** 1 Department I of Internal Medicine, Excellence Centre for Medical Mycology (ECMM), University of Cologne, Faculty of Medicine and University Hospital Cologne, Kerpener Str. 62, 50973 Cologne, Germany; 2 Chair Translational Research, Cologne Excellence Cluster on Cellular Stress Responses in Aging-Associated Diseases (CECAD), University of Cologne, Faculty of Medicine and University Hospital Cologne, Herderstr. 52, 50931 Cologne, Germany; 3 German Centre for Infection Research (DZIF), Partner Site Bonn-Cologne, Herderstr. 52, 50931 Cologne, Germany; 4 Clinical Trials Centre Cologne (ZKS Köln), University of Cologne, Faculty of Medicine and University Hospital Cologne, Gleueler Straße 269, 50935 Cologne, Germany; 5 CoRe Consulting GmbH, Cologne, Germany; 6 Department for Clinical affairs and Crisis management, University of Cologne, Faculty of Medicine and University Hospital Cologne, Kerpener Str. 62, 50973 Cologne, Germany; 7 Healex GmbH, Sophienstraße 5, 51149 Cologne, Germany; 8 Department of Otorhinolaryngology, Head and Neck Surgery, University of Cologne, Faculty of Medicine and University Hospital Cologne, Kerpener Str. 62, 50973 Cologne, Germany; 9 Information Technology uk-it, Medical Applications, University of Cologne, Faculty of Medicine and University Hospital Cologne, Kerpener Str. 62, 50973 Cologne, Germany; 10 Center for Molecular Medicine Cologne (CMMC), Robert-Koch-Str. 21, 50931 Cologne, Germany

**Keywords:** COVID-19 pandemic, SARS-CoV-2 testing strategy, Web-based patient management, Digital medicine

## Abstract

**Background:**

During the SARS-CoV-2 pandemic a mass casualty incident of ambulatory patients occurred at the COVID-19 rapid response infrastructure (CRRI) facility at the University Hospital of Cologne (UHC). We report the development of a patient-centred mobile-device solution to support efficient management of the facility, triage of patients and rapid delivery of test results.

**Methods:**

The UHC-Corona Web Tool (CWT) was developed as a web-based software useable on each patient’s smartphone. It provides, among others, a self-reported medical history including type and duration of symptoms and potential risk contacts and links all retrieved information to the digital patient chart via a QR code. It provides scheduling of outpatient appointments and automated transmission of SARS-CoV-2 test results.

**Results:**

The UHC-CWT was launched on 9 April 2020. It was used by 28,652 patients until 31 August 2020. Of those, 15,245 (53,2%) consulted the CRRI, representing 43,1% of all CRRI patients during the observed period.

There were 8304 (29,0%) specifications concerning travel history and 17,145 (59,8%) indications of ≥1 symptom of SARS-CoV-2 infection. The most frequently indicated symptoms were sore throat (60,0%), headache (50,7%), common cold (45,1%) and cough (42,6%) while 11,057 (40,2%) patients did not report any symptoms. After implementation of the UHC-CWT, the amount of patient contacts per physician rose from 38 to 98,7 per day. The personnel for communication of test results were reduced from four on seven days to one on five days.

**Conclusion:**

The UHC-CWT is an effective digital solution for management of large numbers of outpatients for SARS-CoV-2 testing.

**Supplementary Information:**

The online version contains supplementary material available at 10.1186/s12879-021-06249-7.

## Background

From the end of February 2020 onwards, many patients were seen at the emergency department as well as the COVID-19 rapid response infrastructure (CRRI) facility of the University Hospital of Cologne (UHC) in context of the SARS-CoV-2 pandemic [1]. Extensive prior preparation was not possible. The close geographical proximity to one of Germany’s first SARS-CoV-2 hotspots in the Heinsberg area on top of numerous travel returners from the Alpine region, led to maxed-out capacity [2, 3]. In addition, numerous UHC employees with a relevant travel history or contact with SARS-CoV-2 positive patients had to be tested to avoid further spread of the disease within the hospital system [4, 5].

This mass influx of ambulatory patients demanded a drastic increase of efficiency for up-front triage of patients, consultations and rapid communication of test results while ascertaining constant quality.

To reduce consultation time to a reasonable minimum, a paper-based questionnaire on prior medical history was distributed to waiting patients. The questionnaire was available in several languages and updated on a daily basis to include new findings, such as newly declared high-risk areas or newly identified symptoms of COVID-19 such as olfactory or taste disorders [6–8]. This questionnaire was then scanned and included as source data in the individual electronic patient file.

It soon became evident, that a digital approach was needed to support this overwhelming situation.

## Methods

The following goals were set: enabling patients to take their own medical history, embedding the Web Tool into the IT system of the CRRI and the automated communication of negative SARS-CoV-2-PCR results.

### Development of the UHC Corona web tool

The rapidly changing epidemiological situation required a continuously flexible software development in a limited time frame. Definition of the process flow and legal review of applicable data security and privacy requirements as well as development of texts for automated electronic notifications took place simultaneously. The development of the UHC Corona Web Tool (Healex GmbH, Cologne, Germany) was conducted in accordance with principles of agile software development using the *Scrum* method. The Scrum method entailed the setting of a time frame (so-called *Sprint*), in this case two weeks, in which the product increment was developed. In preparation of the Sprint, requirements were defined and prioritized. In the course of the Sprint, a continuous exchange between development team and users took place. By the end of the Sprint, as many defined requirements as possible should be met and the product should be finalized.

The electronic patient chart regularly used by UHC in the clinical information system (CIS) ORBIS® (Dedalus HealthCare GmbH, Bonn, Germany) and SAP-SE (SAP, Walldorf, Germany) were linked with the patient chart of the UHC Corona Web Tool.

### Utilization of the UHC Corona web tool

**Fig. 1:**
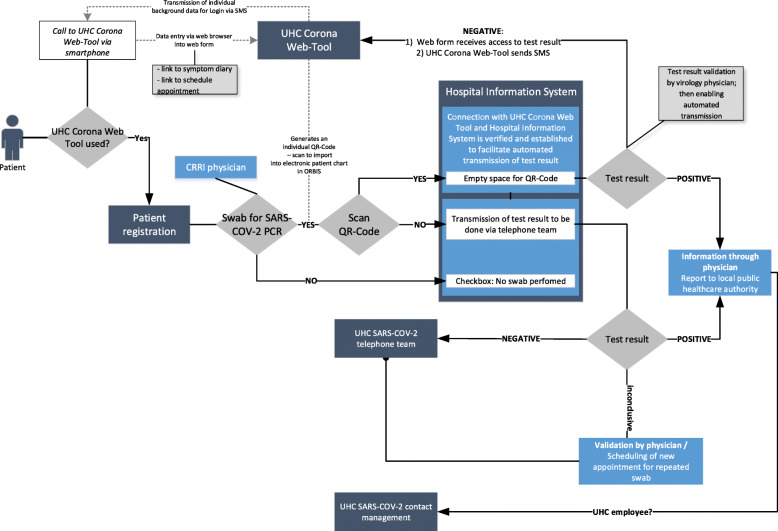
Flow diagram of the organizational structure of the CRRI including UHC Corona Web Tool. CRRI, COVID-19 rapid response infrastructure; UHC, University Hospital Cologne

The UHC Corona Web Tool was announced via internet, signage on the hospital campus as well as the mobile CRRI. A smartphone call to the UHC hotline automatically initiates a SMS with a link being sent to the caller. The link opens the UHC Corona Web Tool on the smartphone browser. Once the patient has agreed to the data privacy terms, he/she can provide his/her personal medical history (Fig. A1). The Web Tool then sends the following information to the caller: 1) travel directions to the CRRI, 2) a link to schedule an appointment 3) a link including a QR-code. At the CRRI site, the QR-code is scanned to transfer the patient-provided medical history to the electronic patient chart in the CIS. (Fig. 1).

All features can be used via any standard internet browser on an ordinary smartphone and do not require installation of an additional app.

### Self-reported medical history and test implementation

The self-reported medical history (MH) conducted through the UHC Corona Web Tool records the type and duration of symptoms, risk contacts, stays in high risk-areas, underlying diseases and prognostic factors. The questionnaire was answered before entering the CRRI.

During the subsequent physician consultation, the patient was supposed to show the MH to the physician who evaluated the indication for SARS-CoV-2 testing via nasopharyngeal swab. Current recommendations of the German Centre for Disease Control, the *Robert Koch Institute* (RKI) were applied, whilst taking into account further epidemiological and clinical criteria [9, 10].

A negative SARS-CoV-2-PCR test result was communicated within seconds via an automatically dispatched SMS right after validation of the PCR test result by a UHC virologist. A data link between the virologic laboratory and the UHC Corona Web Tool allowed for this notification. Positive test results were communicated via a telephone call consultation conducted by a physician (Fig. 1). Additionally, a link to a fourteen-day symptom entry log was sent to the caller to document the potential development of symptoms suggestive of COVID-19. This also included a calendar notification function. The log was developed in accordance with recommendations of the RKI [11].

### Data privacy and ethical statement

The utilization of the UHC Corona Web Tool does not require an external login into a patient file, nor does it require access to a hospital’s IT system. All user interaction is conducted via SMS using the individual’s phone number. No personal information is shared or made available online at any given time. According to the technical state of the art, compromise of the data through third parties is therefore not possible. The informed consent to use anonymized data is actively given by the user during the administrative process in the UHC Web Tool. A revocation of consent is possible via the data safety officer of the UHC.

Informed consent was obtained from all subjects (or if subjects under 18, from a parent and/or legal guardian) regarding the anonymized scientific evaluation of their reported data. This study and all methods were carried out in accordance with the declaration of Helsinki. The data was completely anonymized before analysis, therefore ethics approval by the ethics committee of the University Hospital of Cologne was waived.

### Analysis

The data generated through the implementation of the UHC Corona Web Tool concerning symptoms, risk contacts and SARS-CoV-2 test results was analysed in a descriptive manner. A possible influence of the increased usage of the UHC Corona Web Tool on efficiency in the consultation was assessed by quantitatively analysing the patient contact per physician. The cut-off date of the here presented data is 31 August 2020.

## Results

The first operational version of the UHC Corona Web Tool was completed in one sprint. In a following internal test phase, further requirements were evaluated. After three weeks of development, the Web Tool was launched on 9 April 2020. Since project initiation, the methodology for further development has remained the same. This method entails an update conducted every two weeks regarding additional user functions as well as updates necessitated by the epidemiological situation.

Geographically, the CRRI’s catchment area includes the adjacent neighbourhoods as well as other areas within and around the city of Cologne, Germany. The number of patients per region (postal code and country) can be found in Fig. A2a-d.

Since the establishment of the CRRI, a total of 35,378 ambulatory patients were seen until 31 August 2020 and 36,214 nasopharyngeal swabs for SARS-CoV-2-PCR tests were conducted in this time period. The discrepancy in results is due to multiple testing of some individual patients. On average, 1263 patients per week over the described period were seen. Prior to the implementation of the UHC Corona Web Tool 1150 patients per week were seen. After implementation of the UHC Corona Web Tool, 27,515 patients were seen in the CRRI (1310 per week on average) (Table 1).

**Table 1:** Patient contacts at CRRI per physician per week and day and CRRI SARS-CoV-2 test results

Calendar week	Patient contacts / week (*n*)	CRRI physicians (*n*)	Patients contacts / CRRI physician / week (*n*, rounded)	Patient contacts / CRRI physician / day (mean) (*n*, rounded)	SARS-CoV-2 test positive n	SARS-CoV-2 test positive %
*Before introduction of UHC Corona Web Tool*						
9	124	1	124	18	1	0.81
10	505	2	253	36	8	1.58
11	1366	4	342	48	171	12.52
12	2092	5	418	59	146	6.98
13	1552	6	259	37	147	9.47
14	1263	6	211	30	98	7.76
	6902 (Mean: 1150) (Median 1315)			38 (Median: 36.5)		
*After introduction of UHC Corona Web Tool*						
15	961	6	160	23	79	8.22
16	1076	4	269	38	86	7.99
17	1104	4	276	39	58	5.25
18	1666	4	417	60	41	2.4
19	2322	4	581	83	32	1.38)
20	2052	4	513	73	14	0.68
21^a^	1303	4	326	54	8	0.61
22^b^	1173	2	587	117	7	0.59
23	850	2	425	85	0	0.0
24	708	2	354	71	4	0.56
25	957	2	479	96	6	0.63
26	978	2	489	98	4	0.41
25	1010	2	505	101	11	1.09
28	1145	2	573	115	14	1.22
29	1185	2	593	119	17	1.43
30	1292	2	646	129	20	1.55
31	1476	2	738	148	22	1.49
32	1658	2	829	166	21	1.27
33	1695	2	848	170	20	1.18
34	1642	2	821	164	22	1.34
35	1786	2	893	179	28	1.57
36§	437	2	219	44	3	0.69
	27,515 (Mean: 1310) (Median: 1185)			98.7 (Median: 97)		

Calendar week 15 = UHC Web Tool launched

^a^CRRI opening hours reduced from seven weekdays to six weekdays, ^b^ CRRI opening hours reduced from six weekdays to five weekdays, §Calendar week 36 only included until 31 August 2020

Abbr.: *CRRI* COVID-19 rapid response infrastructure facility; *UHC* University Hospital of Cologne

**Fig. 2:**
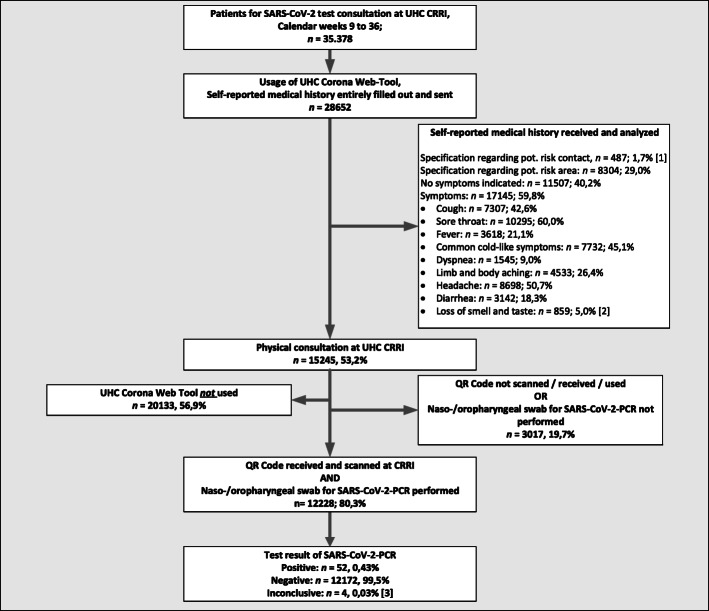
Characteristics of UHC Corona Web Tool users. CRRI, COVID-19 rapid response infrastructure facility; UHC, University Hospital of Cologne. [1] Feature excluded since calendar week 27 due to inaccurate answers. [2] included since May 17th, 2020 (calendar week 28). [3] Inconclusive test results usually lead to a rapid notification of the patient and a repeated appointment for an additional swab for SARS-CoV-2 PCR; two patients did not consult the CRRI again after being notified to do so

The UHC Corona Web Tool was used by 28,652 patients. Of these, 17,145 users (59.8%) filled out the questionnaire on medical history *completely*. This resulted in 8304 (29.0%) data entries on travel information and 487 (1.7%) data entries on potential risk contacts with SARS-CoV-2 positive persons. The latter entry option was excluded from the 27th calendar week onwards due to users’ inaccurate data entry and additional potential confounders. Overall, 17,145 (59.8%) of patients reported symptoms. The most common symptoms were: sore throat (60.0%), headache (50.7%), common flu-like symptoms (45.1%) and cough (42.6%). The remaining 11,057 patients (40.2%) reported no symptoms at all (Fig. 2).

15,245 users of the of the UHC Corona Web Tool (53.2%) visited the CRRI in person. Of these, 12,228 patients (80.3%) underwent SARS-CoV-2-PCR testing via nasopharyngeal swab. 52 tests (0.43%) were positive and 12,172 were negative for SARS-CoV-2 (Fig. 2). Out of the 52 SARS-CoV-2 positive tested patients, none was admitted for inpatient treatment in our hospital.

**Fig. 3:**
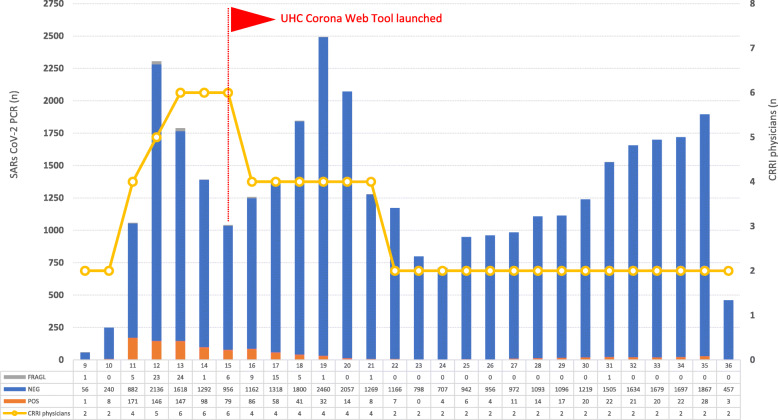
Patient contacts, number of UHC physicians and SARS-CoV-2 test results from naso−/oropharyngeal swabs at CRRI. CRRI, COVID-19 rapid response infrastructure facility; UHC, University Hospital of Cologne, POS, positive SARS-CoV-2 test result; NEG, negative SARS-CoV-2 test result; FRAGL, inconclusive SARS-CoV-2 test result

Prior to the implementation of the UHC Corona Web Tool, the average number of patient contacts per physician per day was 38 with a maximum of 59 patient contacts per physician per week in the 12th calendar week (Tab. 1). After introduction of the UHC Corona Web Tool, the total number of patient contacts in the CRRI per week initially decreased. Also, the number of employed physicians at the CRRI decreased and the opening hours were reduced from seven days to five days a week. In the further course, the number of patient contacts in the CRRI increased, partially due to a public call for intensified testing (Fig. 3). Subsequently, the number of patient contacts per physician per day increased to 98.7 with a maximum of 893 patients per physician per week in the 35th calendar week (Fig. 3 and Table 1).

The communication of test results through the *UHC SARS-CoV-2 contact management team* was also optimized. After introduction of the UHC Corona Web Tool, the personnel required was reduced from four employees in two shifts on seven days to one employee available during a regular working day on five days per week.

## Discussion

The efficient deployment of healthcare personnel plays a decisive role in managing a critical outbreak during the SARS-CoV-2 pandemic [12]. Our study highlights the use of a web-based patient management at a COVID-19 testing facility for patients with mild or no symptoms suggestive for COVID-19. The introduction of the UHC Corona Web Tool supported SARS-CoV-2 testing by increasing patient contacts per physician. Furthermore, the personnel required for the communication of test results was reduced. We report the methodology of an easy-to-use and accessible solution to facilitate triage, testing and result delivery for SARS-CoV-2 testing. We report findings from an analysis of the user data of 28,652 users increasing efficiency from an average patient number per physician per day from 38 up to 98.7 while assuring equal quality of care for patients. Since the COVID-19-specific MH of a patient was already taken, paperwork and documentation were avoided and indication for SARS-CoV-2 swab could be checked within seconds, the physician in charge was able to skip those steps, save time and focus on the patient’s condition.

A variety of apps have been developed to manage the SARS-CoV-2 pandemic [13]. However, app development is lengthy, depending on the used platform and has a range data of regulatory pitfalls. The main difference between apps and our web-based method is the personalized link which identifies the patient just in that single case of admittance. By using a web-based solution we were able to establish a satisfactory process very quickly while meeting all applicable data protection requirements. As a web-based tool, no additional application (APP) needs to be installed. The tool works via a browser and is compatible with almost every device. Web-based approaches to record symptoms and subsequent medical implications in the context of COVID-19 have been developed and validated by other groups [14]. Our approach includes a patient-centred perspective and additionally connects it with several other functions.

The UHC Corona Web Tool enables triage of patients via symptom analysis in the following manner: 1.) Patients with contact to a SARS-CoV-2 positive tested person 2.) symptomatic and 3.) asymptomatic patients. This triage is carried out before the patient enters the CRRI. This procedure prevents possible transmissions of the virus in the CRRI site itself [15, 16]. In addition, the contactless use of smartphones prevents possible infections via touch of surfaces and provided materials such as pens as well as close contact while exchanging these items [17, 18]. Mass testing sites are known to play a key role in the control of pandemics [19]. By connecting the UHC Corona Web Tool to the scheduling of appointments, the CRRI capacity can be optimized. This also facilitates the allocation of required testing materials.

By using a web-based approach, time pressure on patients reporting their medical history is avoided which can increase accuracy and validity of the medical history. Through storage of the fourteen-day-long symptoms log on the patient’s smartphone, the documentation of the development of symptoms is easily accessible and may help to facilitate contact tracing by public healthcare institutions. In line with other research, the most common symptoms suggestive of SARS-CoV-2 infection in the described cohort were: common cold, cough, headache and sore throat [20]. According to the current state of knowledge, these symptoms have the highest sensitivity concerning mild and ambulatory SARS-CoV-2 infections. These symptoms, however, are unspecific and are of low diagnostic relevance [21]. Loss of taste and smell was included after launching the Web Tool, therefore, this symptom is not adequately represented in our analysis [22, 23]. Of note, no patient using the UHC CWT were subsequently admitted for inpatient care in our hospital since patients with severe symptoms suggestive of COVID-19 were not tested in the CRRI but presented immediately to the UHC emergency department for further care.

The percentage of UHC Web Tool users who subsequently underwent SARS-CoV-2 testing was low in comparison to all patients consulted at the CRRI. Especially UHC employees received multiple tests, which led to an increase in the overall rate of tests. These employees, however, did not use the UHC Corona Web Tool as often. It is possible, that the increase in efficiency is due to stricter evaluation regarding test indications updated by the RKI as well as due to the implementation of the UHC Corona Web Tool [9].

Our approach certainly has limitations. The usage of the UHC Corona Web Tool is voluntary. Since older patients do not use smartphones as frequently, the population that uses the UHC Corona Web Tool differs from the groups at risk of severe SARS-CoV-2 infection [24]. In our experience, the quality of care of patients *not* using the UHC Corona Web Tool was not compromised, however, this aspect was not analysed qualitatively in the margin of this study. The UHC Corona Web Tool is continuously updated due to the dynamic of the COVID-19 pandemic. This may limit the structured and comparative analysis of the data in the future, e.g. by adding additional items to the survey and thereby causing biases. The here described data from mid-April to August represents a low incidence period of SARS-CoV-2 in Germany. Lack of internet connection, data leakage and other technical issues could cause lack of utilization of the UHC Corona Web Tool, e.g. patients who presented more than once to the CRRI were sent the same text message concerning a negative test result several times which lead to confusion. This error was resolved within one week.

Software like the UHC Corona Web Tool can provide detailed and anonymized data for analysis of the development of infections in a scientific context [25]. The utilization of a web-based application provides new perspectives for the future of the healthcare system in general and specifically with regard to future pandemics regardless of their aetiology. Add-ons such as automated timely reporting to public healthcare institutions in accordance with any legislation could be implemented [26]. This might further decrease work load and optimize personnel allocation during a critical pandemic situation in the near future. A web-based approach can be part of an emergency strategy for mass testing or part of routine testing to avoid regional lockdowns and thus prevent substantial disruptions in everyone’s daily life. An immediate communication of test results is possible. Therefore, critical time delays in regard to the identification of infection chains are no longer encountered [27].

## Conclusions

The UHC Corona Web Tool facilitated SARS-CoV-2 testing by increasing patient contacts per physician. Also, the personnel required to communicate test results was reduced. Software like the UHC Corona Web Tool can support the collection of data to analyse developments in infectious diseases. Web-based applications open up new perspectives for our future healthcare systems, especially in regard to future pandemics. The UHC Corona Web Tool represents an effective digital solution for the overall management from admission right through to the test result for large scale testing of ambulatory patients during the COVID-19 pandemic.

## Supplementary Information

**Additional file 1 Fig. A1**. Exemplary screenshot of UHC Corona Web Tool (in German). **Fig. A2a-d**. Distributions of postal codes (place of residence) of outpatients of the UHC CRRI from February 25th until August 31st, 2020.

## Data Availability

All data, materials and software application comply with field standards regarding data transparency. The datasets analyzed are available from the corresponding author upon reasonable request.

## Abbreviations

CCRI: COVID-19 rapid response infrastructure
CIS: Clinical information system
CWT: Corona Web Tool
RKI: Robert Koch Institute
UHC: University Hospital of Cologne
