# Supplementary material for: Web-based, rapid and contactless management of ambulatory patients for SARS-CoV-2-testing
Source: BMC Infect Dis. 2021 Jun 7;21:535. doi: 10.1186/s12879-021-06249-7 (PMC8182346; doi:10.1186/s12879-021-06249-7)

Web-based, rapid and contactless management of ambulatory patients for SARS-CoV-2-testing

Supplementary Material

Jannik Stemler[1,2,3], Oliver A. Cornely[1,2,3,4,5], Torsten Noack-Schönborn[6], Corinna Fohrholz[7], Sofie Schumacher[1,2], Leonard Poluschkin[8], Bernd Binder[9], Clara Lehmann[1], Georg Langebartels[6]

1. University of Cologne, Faculty of Medicine and University Hospital Cologne, Department I of Internal Medicine, Excellence Centre for Medical Mycology (ECMM), Kerpener Str. 62, 50973 Cologne, Germany
2. University of Cologne, Faculty of Medicine and University Hospital Cologne, Chair Translational Research, Cologne Excellence Cluster on Cellular Stress Responses in Aging-Associated Diseases (CECAD), Herderstr. 52, 50931 Cologne, Germany
3. German Centre for Infection Research (DZIF), Partner Site Bonn-Cologne, Herderstr. 52, 50931 Cologne, Germany
4. University of Cologne, Faculty of Medicine and University Hospital Cologne, Clinical Trials Centre Cologne (ZKS Köln), Gleueler Straße 269, 50935 Cologne, Germany
5. CoRe Consulting GmbH, Cologne, Germany
6. University of Cologne, Faculty of Medicine and University Hospital Cologne, Department for Clinical affairs and Crisis management, Kerpener Str. 62, 50973 Cologne, Germany
7. Healex GmbH, Sophienstraße 5, 51149 Cologne, Germany
8. University of Cologne, Faculty of Medicine and University Hospital Cologne, Department of Otorhinolaryngology, Head and Neck Surgery, Kerpener Str. 62, 50973 Cologne, Germany
9. University of Cologne, Faculty of Medicine and University Hospital Cologne, Information Technology uk-it, Medical Applications, Kerpener Str. 62, 50973 Cologne, Germany

Corresponding author
Georg Langebartels, MD
Department for Clinical affairs and Crisis management
University Hospital of Cologne
Kerpener Str. 62
50973 Cologne
phone: +49 221 478-86980
email: georg.langebartels@uk-koeln.de

Fig. A1. Exemplary screenshot of UHC Corona Web Tool (*in German*)

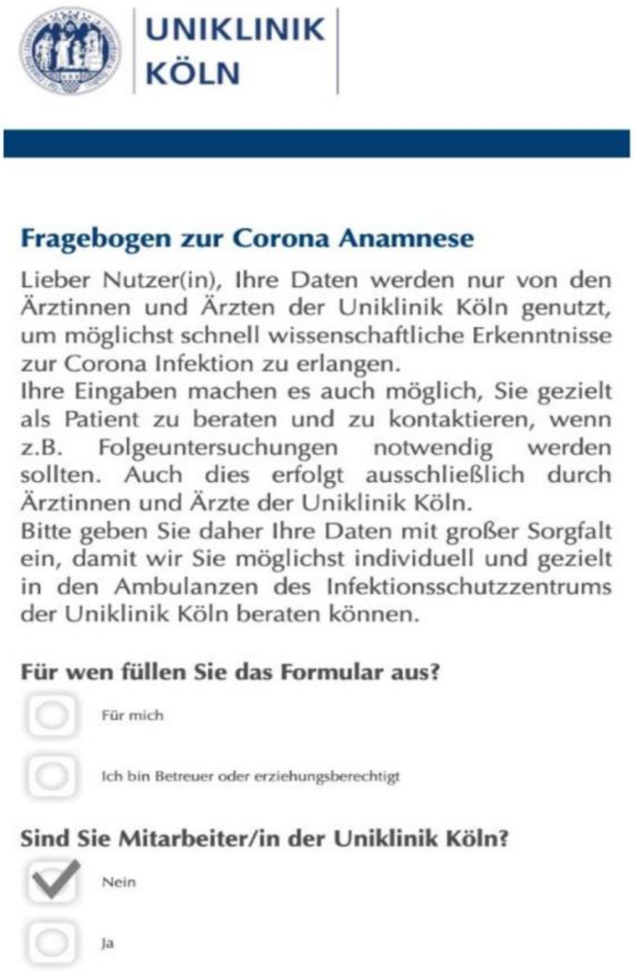

UNIKLINIK KÖLN

Fragebogen zur Corona Anamnese

Lieber Nutzer(in), Ihre Daten werden nur von den Ärztinnen und Ärzten der Uniklinik Köln genutzt, um möglichst schnell wissenschaftliche Erkenntnisse zur Corona Infektion zu erlangen.
Ihre Eingaben machen es auch möglich, Sie gezielt als Patient zu beraten und zu kontaktieren, wenn z.B. Folgeuntersuchungen notwendig werden sollten. Auch dies erfolgt ausschließlich durch Ärztinnen und Ärzte der Uniklinik Köln.
Bitte geben Sie daher Ihre Daten mit großer Sorgfalt ein, damit wir Sie möglichst individuell und gezielt in den Ambulanzen des Infektionsschutzzentrums der Uniklinik Köln beraten können.

Für wen füllen Sie das Formular aus?

- ( ) Für mich
- ( ) Ich bin Betreuer oder erziehungsberechtigt

Sind Sie Mitarbeiter/in der Uniklinik Köln?

- (✓) Nein
- ( ) Ja

Fig. A2. Distributions of postal codes (place of residence) of outpatients of the UHC CRRI from February 25th until August 31st, 2020

a. Cologne City Area

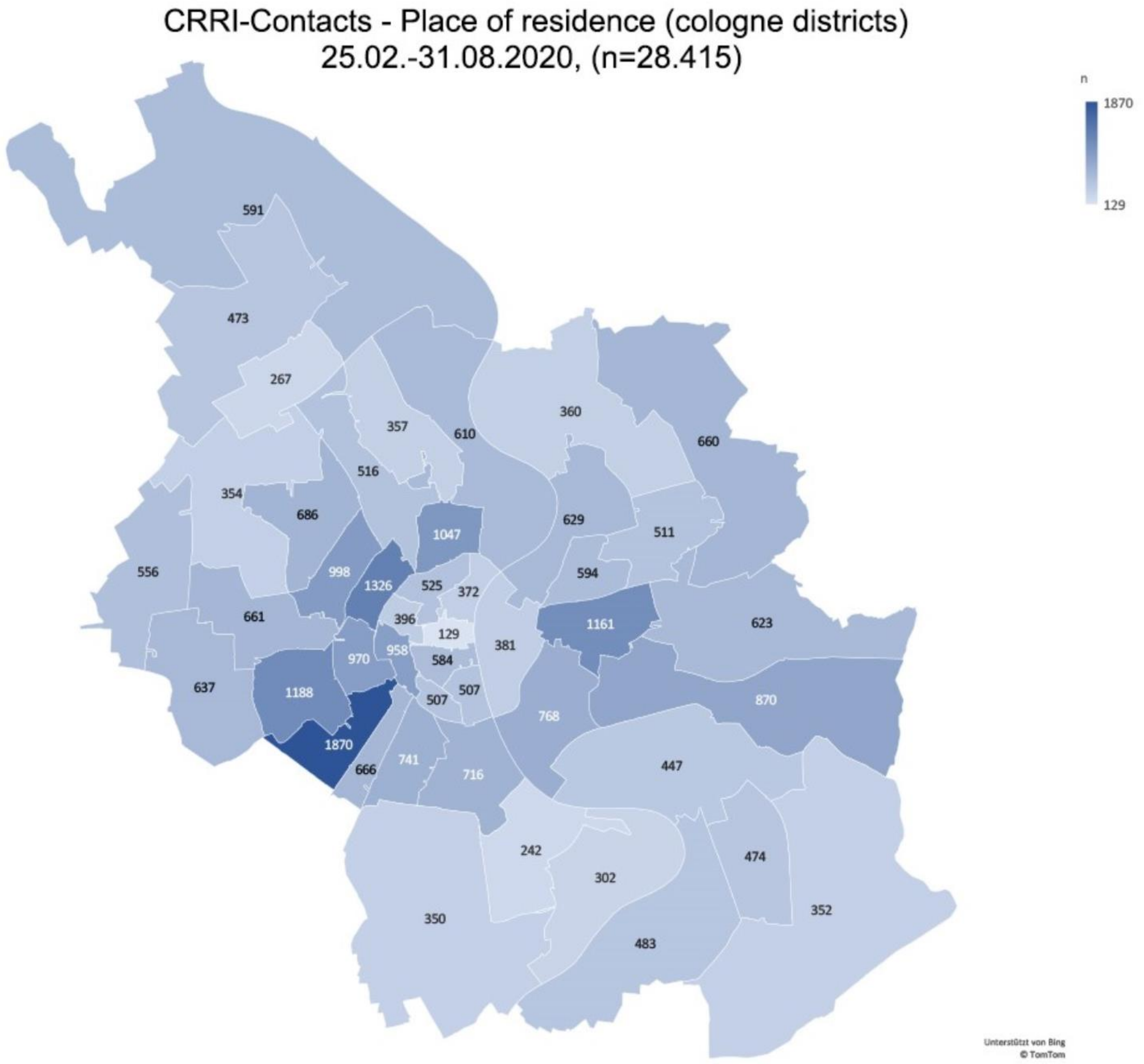

b. City of Cologne greater Regional Area

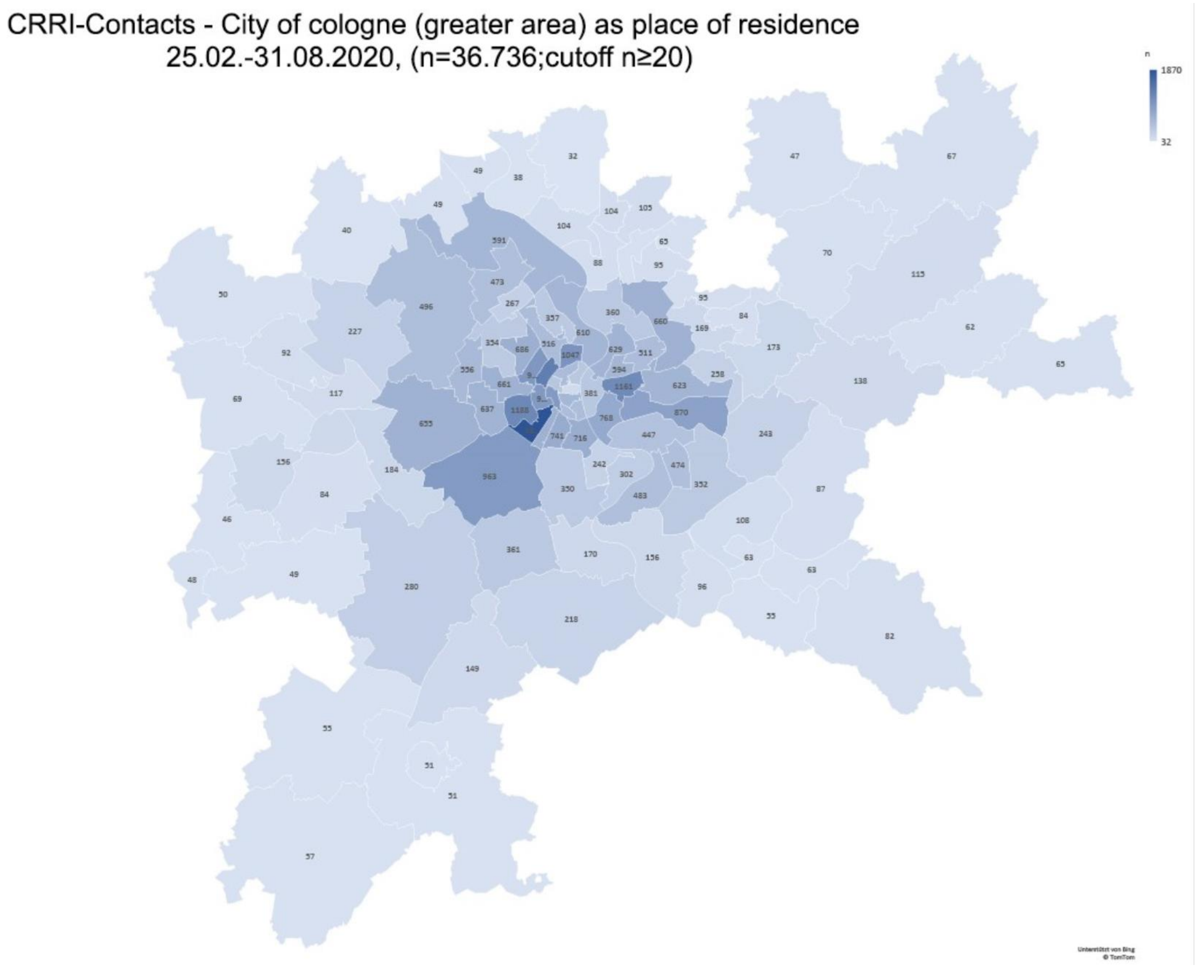

c. Germany

CRRI contacts – place of residence (Germany-wide)

25.02.-31.08.2020

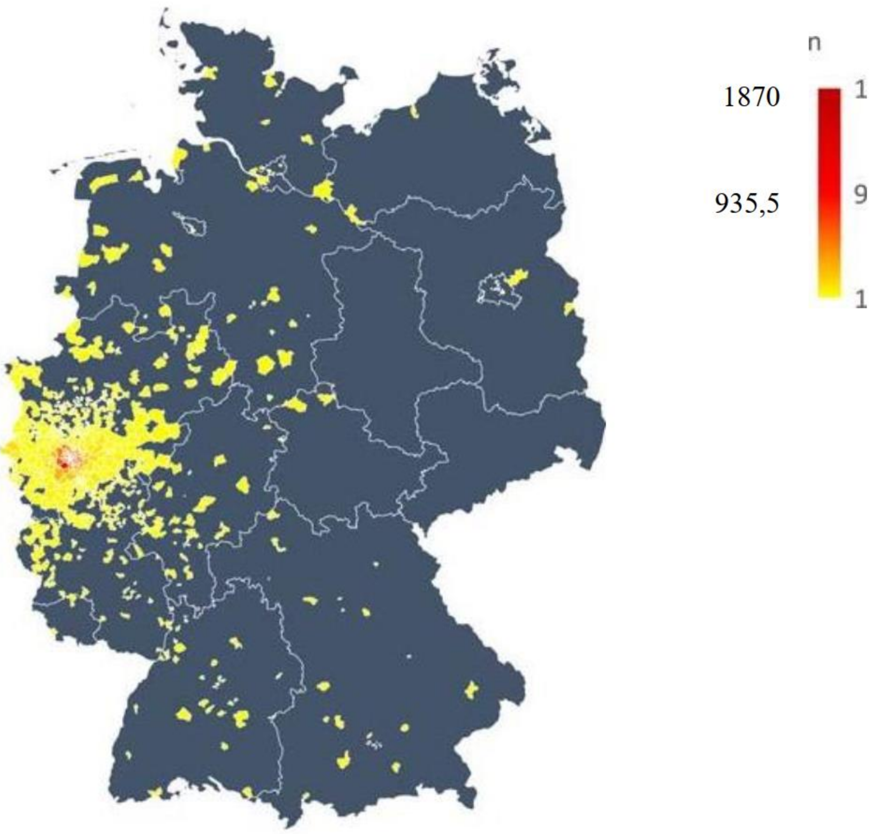

d. Worldwide / history of foreign travel (except Germany)

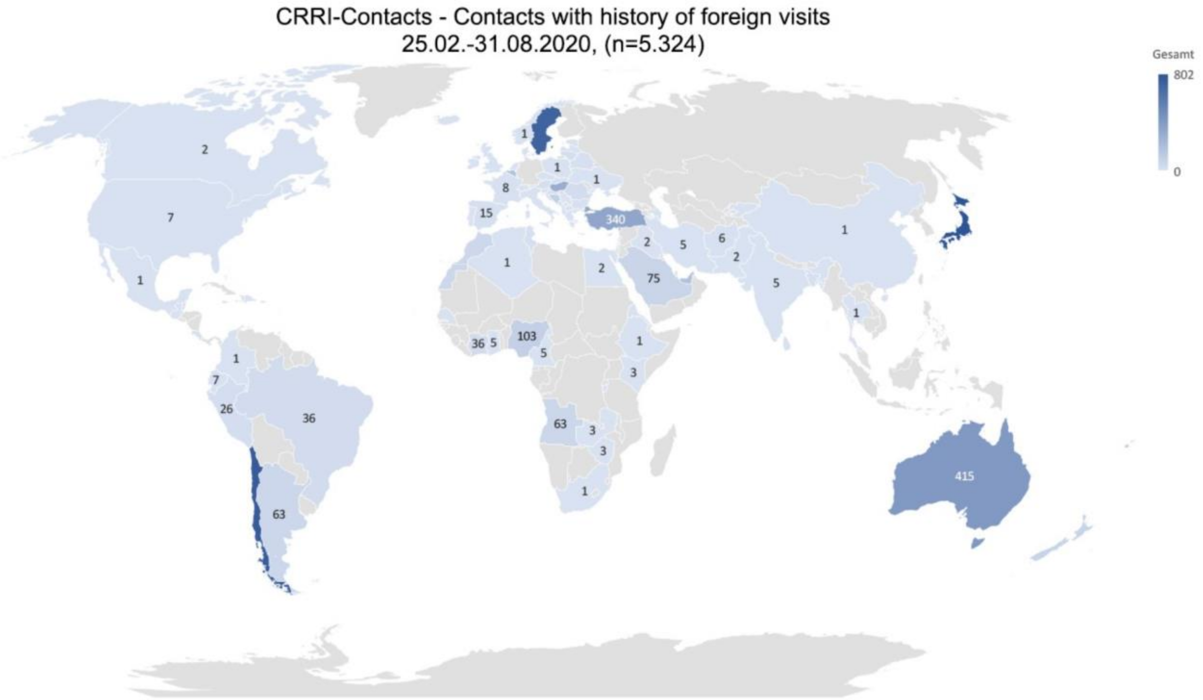

Supplement: Supplementary file 1 — Additional file 1 Fig. A1. Exemplary screenshot of UHC Corona Web Tool (in German). Fig. A2a-d. Distributions of postal codes (place of residence) of outpatients of the UHC CRRI from February 25th until August 31st, 2020. [file 12879_2021_6249_MOESM1_ESM.pdf]
